# A Nonenzymatic Glucose Sensor Platform Based on Specific Recognition and Conductive Polymer-Decorated CuCo_2_O_4_ Carbon Nanofibers

**DOI:** 10.3390/ma13122874

**Published:** 2020-06-26

**Authors:** Yongling Ding, Huadong Sun, Chunrong Ren, Mingchen Zhang, Kangning Sun

**Affiliations:** 1 School of Transportation Civil Engineering, Shandong Jiaotong University, Jinan 250357, China; 214052@sdjtu.edu.cn; 2 School of Control Science and Engineering, Shandong University, Jinan 250002, China; 3 Postdoctoral Technology Research Center, Shandong Anran Group, Weihai 264205, China; zhangmingchen@126.com; 4 School of Materials Science and Engineering, Shandong University, Jinan 250002, China

**Keywords:** electrospinning, CuCo_2_O_4_, thiophene-3-boronic acid, electrocatalytic activity, glucose sensor

## Abstract

CuCo_2_O_4_ decoration carbon nanofibers (CNFs) as an enzyme-free glucose sensor were fabricated via electrospinning technology and carbonization treatment. The CNFs with advantages of abundant nitrogen amounts, porosity, large surface area, and superior electrical conductivity were used as an ideal matrix for CuCo_2_O_4_ decoration. The resultant CuCo_2_O_4_–CNF hybrids possessed favorable properties of unique three-dimensional architecture and good crystallinity, accompanied by the CuCo_2_O_4_ nanoparticles uniformly growing on the CNF skeleton. To further enhance the selective molecular recognition capacity of the developed sensor, a conductive film was synthesized through the electropolymerization of thiophene and thiophene-3-boronic acid (TBA). Based on the synergistic effects of the performances of CNFs, CuCo_2_O_4_ nanoparticles, and boronic acid-decorated polythiophene layer, the obtained poly(thiophene-3-boronic acid) (PTBA)/CuCo_2_O_4_–CNF-modified electrodes (PTBA/CuCo_2_O_4_–CNFs/glassy carbon electrode (GCE)) displayed prominent electrocatalytic activity toward electro-oxidation of glucose. The fabricated sensor presented an outstanding performance in the two linear ranges of 0.01–0.5 mM and 0.5–1.5 mM, with high selectivity of 2932 and 708 μA·mM^−1^·cm^−2^, respectively. The composite nanofibers also possessed good stability, repeatability, and excellent anti-interference selectivity toward the common interferences. All these results demonstrate that the proposed composite nanofibers hold great potential in the application of constructing an enzyme-free glucose sensing platform.

## 1. Introduction

Due to a significant number of health concerns bringing about poor diet and environmental degradation, people are increasingly concerned about the detection of diseases such as diabetes, high blood pressure, and cancer. Diabetes is one of the diseases that pose a great threat to human health. For many diabetic patients, how to monitor the blood glucose level quickly, in real time, and accurately is of great significance in the diagnosis and treatment of diabetes [1]. In past glucose detection studies, chemiluminescence, colorimetry, high-performance liquid chromatography, fluorescence, etc. proved to be effective detection methods [2]. Presently, glucose oxidase (GOD)-based glucose biosensors are widely known for their outstanding sensitivity, selectivity, and low detection limit [3]. However, due to the significant detection performance of enzyme biosensors, most commercial glucose sensors are primarily based on glucose oxidase (GOx). However, due to the intrinsic properties of enzymes such as chemical and thermal instability, these inevitable defects and complicated manufacturing procedures may limit their extensive analytical applications. In comparison with enzymatic-based glucose sensors, non-enzymatic-based sensors are receiving increasing attention because of their apparent advantages of sensitive performance, accessible modification approach, and high stability [4]. Meanwhile, sensitivity, selectivity, and anti-interference as critical factors determine the performance of enzyme-free sensors. Based on a large number of previous studies, various precious metals or their alloys were widely explored for the progress and research of enzymatic free sensors (i.e., Au, Pt, Pd, Ru, PtPd, PtCu, PtAu, etc.) [5], due to their remarkable catalytic activities and outstanding sensitivities toward glucose oxidation. However, there is significant interference when glucose is detected via noble metal-based catalysts, since most physiological coexisting substances (ascorbic acid (AA), NaCl, etc.) can also produce an electrochemical response within the potential range of glucose oxidation. Moreover, it is easy to cause noble metal electrode poisoning due to the adsorption of chloride ions or glucose reaction intermediates. Meanwhile, high cost and resource shortages are stopping their large-scale applications in catalysis fields.

Recently, in place of traditional precious metals, electrode modification materials based on transition metal oxides (Co_3_O_4_, NiO, CuO, Cu_2_O, etc.) were proposed, as they have the characteristic of unfilled *d*-orbitals and unpaired *d*-electron special electronic structures, which could form a stable multi-oxidation system and have excellent direct electrocatalytic activity [6,7,8]. As for the various transition metal oxides, binary metal oxides (such as MnCo_2_O_4_ [9], NiFe_2_O_4_ [10] CuMn_2_O_4_ [11], NiCo_2_O_4_ [12], etc.) are widely used in electrochemical catalytic oxidation of glucose because of their better stability in air and solution, non-self-poisoning phenomenon, excellent sensitivity, and selectivity in comparison with noble metals. Moreover, bimetallic oxides also exhibit superior electrical conductivity and electro-catalytic properties in contrast with monometallic oxides, attributed to the synergetic enhancement action of the different structure components, as well as the relatively low activation energy between multiple metal cations in the process of electrons transport. In particular, the spinel CuCo_2_O_4_ nanostructure, as the most common ingredient of cobaltite, attracted considerable attention. As well known, the electro-catalytic performance depends on the structure of catalysts; therefore, spinel CuCo_2_O_4_ nanostructures with various shapes, including nanowires, nanoparticles, and nanocubes [13,14,15], were synthesized, and the fabricated electrode materials were applied in various areas, such as Li-ion batteries, catalyst, supercapacitors, etc. However, they still have to face some limitations, such as unsatisfactory conductivity, large brittleness, easy self-agglomeration on the electrode surface, etc., which hinders their application in electrochemical sensors [16]. Recently, it was proven to be an effective strategy to prominently enhance glucose-sensing performance when combining CuCo_2_O_4_ with carbon-based materials [17]. This is mainly because carbon-based materials have the advantages of a large specific surface area, high temperature resistance, and outstanding electrical conductivity, which facilitates a fast electron diffusion rate and provides an adequate interface contact between the modified electrode and the electrolyte [18]. Therefore, by combining CuCo_2_O_4_ materials with different conductive carbon-based frameworks, the hybrid materials could realize the synergistic enhancement of various electrochemical properties.

In particular, in comparison with other carbon supports [19,20,21], one-dimensional (1D) carbon fibers are regarded as a desired matrix material for dispersing active sites because of their attractive structure and electrical/mechanical performance [22]. Meanwhile, electrospinning has a broad application as a valuable and versatile method for the mass production of long polymer fibers with various precursors, with diameters ranging from tens of nanometers to a few micrometers. For electrospinning, by carefully selecting solvents, polymers, and metal precursors, a uniform mixture solution can be formed and then electrospun into composite fibers. Metal salts/polymer precursor fibers could also be converted into metal nanoparticles (NPs) or metal oxide-doped carbon fibers via pyrolysis treatment under an inert atmosphere. More importantly, loading and embedding metal or metal oxides in one-dimensional carbon fibers results in a three-dimensional mesh membrane with many exciting properties, including a large surface-area-to-volume ratio, good mechanical strength, high porosity, and desirable electrical conductivity. All of these factors prove that the electrospun 1D fibrous structure has great potential as a promising support for the decoration of electrocatalytic functional materials to fabricate sensor interfaces through improving the electron transportation or collection efficiency along its long axis, as well as facile functionalization. Additionally, the 1D nanostructure of carbon nanofibers (CNFs) plays a crucial role as a conductive pathway of CuCo_2_O_4_, accordingly decreasing the charge diffusion path and enlarging the contact area of CuCo_2_O_4_ with the electrolyte, which is conducive to the improvement of the electrocatalytic activity. Therefore, the development of CuCo_2_O_4_–CNF hybrids with excellent performance has crucial practical application value.

To fabricate an efficient electrochemical sensor, excellent electrical conductivity and a selective interaction between electrode materials and analytes are crucial. Molecular recognition group combination with conducting polymers provides a suitable strategy for constructing the high-performance biodevices. Boronic acid group-containing compounds, as a synthetic molecular receptor, possess the function of selectively recognizing *cis*-diol configurations in saccharide structures via forming a reversible covalent complex [23].

In critical and extensive research on conductive polymer materials, polythiophene was reported as a promising integrated nanomaterial with the properties of chemical structure diversity, adjustable conductivity, and structural flexibility, and it attracted tremendous attention recently in the preparation of biosensing devices [24,25]. Consequently, boronic acid incorporated with a polythiophene derivative, poly(thiophene-3-boronic acid) (PTBA), which has both outstanding conductivity and biometric performance, could be applied as an identification part for the selective detection of saccharides in a solution.

Herein, in order to obtain a three-dimensional porous structure and highly conductive mixed conjugated structure, CuCo_2_O_4_ nanoparticles uniformly decorated on carbon nanofibers could be synthesized through a facile electrospinning method and subsequent calcination. The bio-identified conductive polymer, PTBA film, could be directly obtained via in situ polymerizations of thiophene-3-boronic acid monomers on CuCo_2_O_4_-modified nanofibers. The three-dimensional porous structure of CuCo_2_O_4_–CNFs is beneficial to the enhancement of the charge transport efficiency and surface area available for the electrocatalytic reaction, while the phenylboronic acid group can be used as a recognition molecule on the electrode surface for the pre-deposition of glucose. Attributed to the synergistic effects of these properties, the resultant hybrid electrode has good electrocatalytic capacity toward glucose, which provides a promising candidate for the construction of efficient enzyme-free sensors.

## 2. Materials and Methods

### 2.1. Materials and Reagents

Polyacrylonitrile (PAN) (molecular weight (Mw) = 150,000), sodium hydroxide (NaOH), cupric acetate monohydrate (Cu(CH_3_COO)_2_·H_2_O), sodium chloride (NaCl), cobalt(II) acetate tetrahydrate (Co(CH_3_COO)_2_·4H_2_O), d-glucose, ascorbic acid (AA), uric acid (UA), l-lysine, dopamine (DA), and *N*,*N*-dimethylformamide (DMF) were provided by Sinopharm Chemical Reagent Corporation (China). Nafion (5 wt.%) was obtained from Sigma-Aldrich. Thiophene, thiophene-3-boronic acid monolayer acid (TBA), and sodium fluoride (NaF) were purchased from Macklin Chemical Reagent Corporation (Shanghai, China).

### 2.2. Preparation of CuCo_2_O_4_-Decorated Carbon Nanofibers (CuCo_2_O_4_–CNFs)

**Scheme 1:**
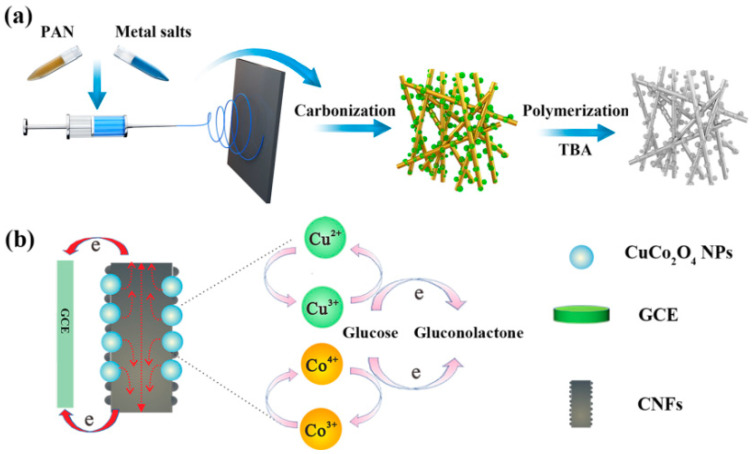
(**a**) Schematic diagram for the preparation of CuCo_2_O_4_–carbon nanofibers (CNFs) and (**b**) the representation of the proposed mechanism for electrocatalytic oxidation of glucose based on poly(thiophene-3-boronic acid) (PTBA)/CuCo_2_O_4_–CNFs/glassy carbon electrode (GCE).

The synthetic procedure for CuCo_2_O_4_–CNFs is illustrated in Scheme 1a. Typically, a spinning precursor solution was obtained by dissolving polyacrylonitrile (PAN, 0.8 g) in 10 mL of *N*,*N*-dimethylformamide (DMF) under constant magnetic stirring at 60 °C for 60 min. Subsequently, the resultant PAN solution was mixed with 0.28 g of copper acetate and 0.66 g of cobalt acetate, and then stirred for 1 h to obtain a homogeneous solution. As for a typical electrospinning process, the obtained precursor solution was transferred into a syringe with an injection rate of 1 mL/h and a voltage of 16 kV. The distance between the spinneret (inner diameter of 0.6 mm) and the drum collector was 15 cm. The humidity and temperature of the electrospinning surroundings were 25% and 25 °C, respectively. As a control, pure PAN fibers (10 wt.% PAN in DMF) were prepared via a similar process. The resulting electrospun nanofibers (NFs) were dried at 60 °C overnight. The corresponding samples were marked as PAN NFs and PAN@CuCo_2_O_4_ NFs, respectively. For carbonization, the stabilization of the obtained nanofiber membrane was conducted at 270 °C for 1 h. Then, the resultant samples were carbonized at 700 °C for 2 h in a nitrogen atmosphere. As for the stabilization and carbonization processes, the controlled heating rates were set as 2 °C·min^−1^ and 5 °C·min^−1^, respectively. After calcination, the transition metal-based nanoparticles were completely converted into CuCo_2_O_4_ by heating the synthesized nanofibers to 300 °C in air for 2 h. The pure carbon nanofibers (CNFs) could be obtained via the same treatment with PAN NFs.

### 2.3. Fabrication of Carbon Nanofibers (CNFs) and CuCo_2_O_4_–CNF-Modified Electrode

Prior to the modification, a glassy carbon electrode (GCE, 3 mm) was surface-polished with alumina slurry and washed with deionized water and ethanol to remove the surface impurities. A homogeneous suspension was obtained by dispersing the prepared nanofibers (2 mg·mL^−1^) with a mixture of 0.02 mL of 1 wt.% nafion and 0.18 mL of DMF, followed by sonication for 60 min to disperse the nanofibers thoroughly. Then, a 10-µL suspension was coated onto the GCE surface, and the corresponding samples were marked as CNFs/GCE and CuCo_2_O_4_–CNFs/GCE, respectively.

### 2.4. Fabrication of PTBA/CuCo_2_O_4_–CNFs/GCE

The poly(thiophene-3-boronic acid) (PTBA) film was prepared by electrochemical polymerization in a solution containing thiophene (25 mM), TBA (25 mM), and 0.2 M NaF in 0.5 M HCl (Scheme 1a). The cyclic voltammetry (CV) detection was conducted with potential set between −0.4 and 1.8 V at a scan rate of 100 mV·s^−1^ in the unstirred solution. Then, the polymer film was rinsed with distilled water and dried in the air. To ascertain the redox behavior of the copolymer film, CV measurement was detected by applying a potential setting of 0.1 to 1.0 V in a 0.5 M HCl solution. After the redox reaction was determined to be stable, the oxidized PTBA films were applied for further electrochemical detection. The resultant sample was labeled as PTBA/CuCo_2_O_4_–CNFs/GCE.

### 2.5. Characterizations

Field-emission scanning electron microscope (FE-SEM, FEI, Hillsboro, OR, USA) measurements were collected on an FEG Quanta 200 FEI and applied to study the surface structures and composition of the samples. X-ray diffraction (XRD) spectroscopy was carried out on an X-ray diffractometer (Rigaku 2550, Rigaku, Tokyo, Japan,) using Cu Kα radiation to record the crystallographic phases of the obtained products. Resonant Raman spectra were conducted on a Renishaw in Via spectrometer with the excitation source of a 514-nm laser. The sample composition and elemental state were measured using X-ray photoelectron spectroscopy (XPS) (Thermo ESCALAB 250, Thermo Fisher Scientific, Waltham, Ma, USA) with Al Kα as the excitation source.

### 2.6. Electrochemical Characterization

All electrochemical behaviors of synthesized nanofibers were investigated on a CHI 660D workstation (Chenhua, Shanghai, China) with a standard three-electrode system. Ag/AgCl, platinum wire, and a modified GCE were selected as the reference electrode, counter electrode, and working electrode, respectively. CV measurements were carried out in 0.1 M NaOH electrolytes between −0.2 and 0.8 V with various scanning rates of 20–200 mV·s^−1^. The differential pulse voltammetry (DPV) parameters were set as pulse amplitude of 50 mV, pulse width of 50 ms, and scanning speed of 20 mV/s. The modified GCE electrode was placed in a glucose solution (10 mM) and stirred for 10 min. The pre-concentrated (accumulated) process of glucose molecules onto the modified electrode was performed in the open circuit. Then, the prepared electrode was washed for electrochemical measurements.

## 3. Results

### 3.1. Structure Characterizations of CNFs and CuCo_2_O_4_–CNFs

**Figure 1:**
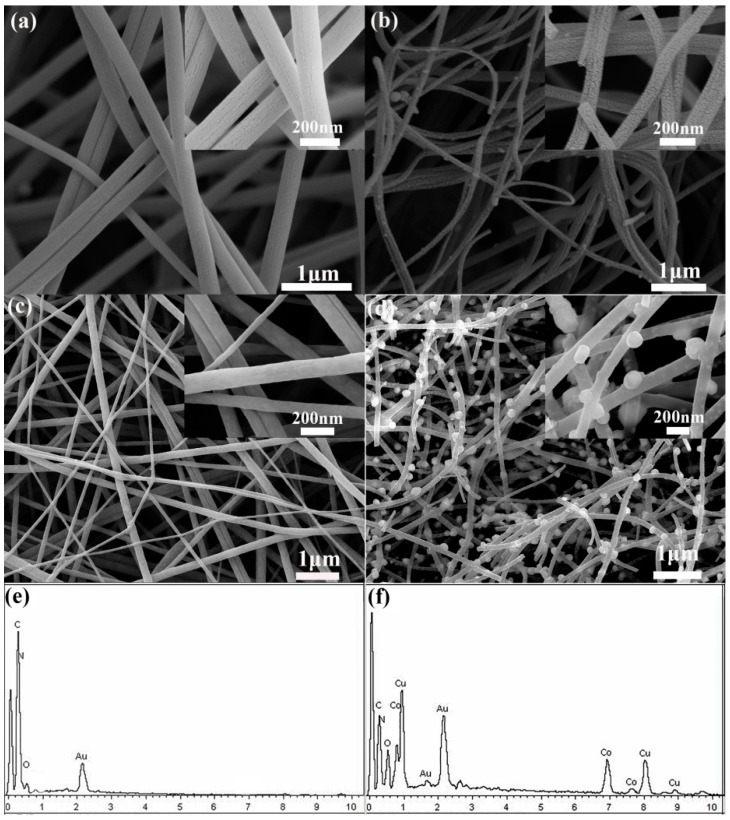
SEM images of (**a**) polyacrylonitrile (PAN) nanofibers (NFs), (**b**) CNFs, (**c**) PAN@CuCo_2_O_4_ NFs, and (**d**) CuCo_2_O_4_–CNFs; insets are the enlarged SEM images of the corresponding samples. Panels (**e**) and (**f**) correspond to the EDX spectrum of (**b**) and (**d**).

The surface morphology and microstructure properties of the as-prepared PAN NFs, PAN@CuCo_2_O_4_ NFs, CNFsm and CuCo_2_O_4_–CNFs are illustrated in Figure 1. The as-spun PAN NFs (Figure 1a) and PAN@CuCo_2_O_4_ (Figure 1c) nanofibers displayed smooth surfaces and 1D nanostructure, with diameters ranging from 100–200 nm and hundreds of micrometers in length. After calcination, the average diameter of CNFs (Figure 1b) and CuCo_2_O_4_–CNFs (Figure 1d) was reduced, whereas they still retained the original nanofiber feature. The size reduction of the nanofibers after annealing was due to the loss of polymer components in the process of annealing. Meanwhile, because of the collapse of the surface structure of the fiber, the smooth surface of fiber morphology became rough after annealing treatment. Moreover, the displayed surface of CuCo_2_O_4_–CNFs was much rougher than that of CNFs. It is well known that a rough surface is favorable to the diffusion of ions and enlarges the interface contact with the electrolyte in the electrochemical reaction process. Several bright nanoparticles with a size of about 100 nm (Figure 1d) were randomly distributed on the surface of CuCo_2_O_4_–CNFs, which were the main decomposition products of cobalt acetate and copper acetate. Both CNFs and CuCo_2_O_4_–CNF composite nanofibers revealed a porous three-dimensional network structure, which was favorable for electron transport and provided sufficient active sites for electrocatalytic reactions. According to the presented elemental analysis shown in Figure 1e,f evaluated by EDX (Energy Dispersive X-ray) spectrum, the presence of C, N, and O elements in CNFs (Figure 1e) and the existence of C, O, N, Co, and Cu elements in CuCo_2_O_4_–CNF composite nanofibers (Figure 1f) ensured the chemical constituents of corresponding nanofibers, which preliminarily confirmed the existence of CuCo_2_O_4_ nanoparticles decorated on the surface of the nanofiber. Nitrogen can be observed in both nanofibers, mainly due to the thermal decomposition of PAN after carbonization. As well known, nitrogen doping could significantly improve the electrical conductivity of carbon-based materials, which is conducive to the rapid transmission/collection of electrons during electrocatalytic reactions [3]. Thus, we believe that the prepared CNFs can be a desirable carrier for CuCo_2_O_4_ nanoparticle decoration, and the synergistic effect of CNFs and CuCo_2_O_4_ is beneficial to enhance the sensitivity of glucose detection.

**Figure 2:**
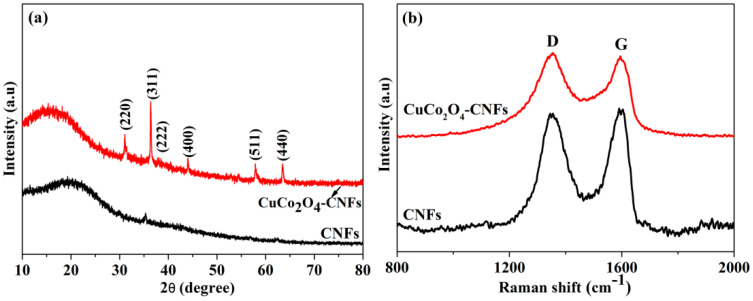
X-ray diffraction (XRD) (**a**) and Raman spectra (**b**) of CNFs (black) and CuCo_2_O_4_–CNFs (red).

XRD, as a valuable tool, could further confirm the composition of prepared CNFs and CuCo_2_O_4_–CNFs. As shown in Figure 2a, a broad diffraction peak around 23° was observed on the XRD pattern of CNFs, which corresponds to graphite, demonstrating the local graphitization feature of the carbon material (JCPDS no. 13-0148) [26]. As for CuCo_2_O_4_–CNFs, in addition to the amorphous carbon peak, all the observed diffraction peaks could be assigned to the CuCo_2_O_4_ phase (JCPDS no. 1-1155).

To further clarify the structural features, the obtained CuCo_2_O_4_–CNF samples were characterized by Raman spectroscopy in the range of 800 to 2000 cm^−1^. The G band commonly represents the plane displacement of carbon atoms in the aromatic structure, while the D band is mainly due to the defective vibration of disordered graphite [27]. In Figure 2b, two distinct Raman peaks were observed on both samples relating to the defect-induced D band and graphitic G band [28]. These results demonstrate that the electrospun PAN polymer fiber is completely converted into conductive CNFs. As an effective standard for the degree of graphitization of the test sample, the I_D_/I_G_ ratio (peak intensity ratio) of CuCo_2_O_4_–CNFs and CNFs was 1.82 and 1.39, respectively, indicating that the order of the graphite phases in CuCo_2_O_4_–CNFs was much higher. Moreover, it was reported that the more ordered graphite structure in carbon materials is favorable to the improvement of electrode conductivity and beneficial to fast electron transportation/collection [29].

**Figure 3:**
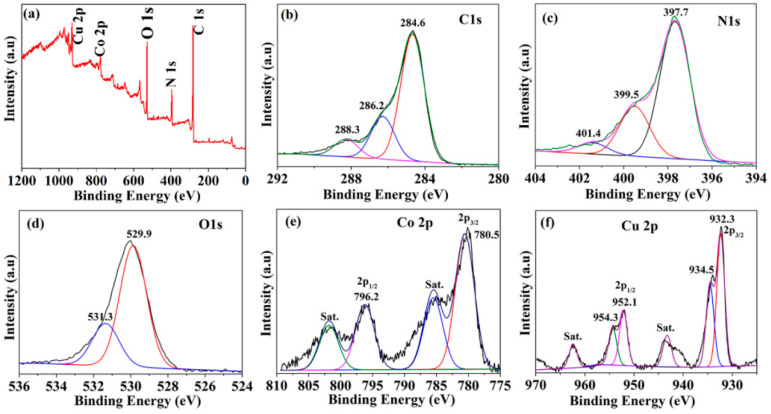
Wide-scan (**a**) and high-resolution X-ray photoelectron spectroscopy (XPS) spectra of CuCo_2_O_4_–CNFs at binding energies corresponding to (**b**) C 1*s*, (**c**) N 1*s*, (**d**) O 1*s*, (**e**) Co 2*p*, and (**f**) Cu 2*p*.

To acquire more information about the obtained hybrid nanofiber, XPS measurements were performed to further study their surface structure and the elemental status of the component elements. The wide-scan spectrum (0–1200 eV) shown in Figure 3a confirmed the peaks of five elements coexisting in the CuCo_2_O_4_–CNFs. In Figure 3b, the three peaks corresponding to the binding energy values around 284.6 eV, 286.2 eV, and 288.3 eV were assigned to the carbon in the form of C–C, C–O, and C=O, respectively [30,31]. As illustrated in Figure 3c, the N 1*s* spectra could be deconstructed into three different chemical states of pyridinic N (397.7 eV), pyrrolic N (399.5 eV), and graphitic N (401.4 eV), implying that N atoms were doped in different positions of the carbon framework [32]. It is worth noting that a certain amount of pyrrolic nitrogen at the edge of the carbon structure provided additional electrons that effectively increased charge mobility and donor–acceptor performances, which could be conducive to the enhancement of conductivity [33]. Therefore, nitrogen-doped carbon nanofibers had the promise of exhibiting better electrocatalytic properties due to the generation of more electrons and the introduction of more defects, as reported in the literature [34]. In Figure 3d, the binding energy peaks located at 529.9 and 531.3 eV correspond to the O 1*s* region. The former peak was assigned to the surface oxygen on the composite surface or adsorbed oxygen species, such as hydroxyl groups, etc., while the latter was mainly due to the metal oxygen bonding formed with the transition metal (with Cu and Co) or the formed lattice O^2−^ bond [35,36]. As for the Co 2*p* spectrum, the two peaks at binding energies of 780.5 and 796.2 eV could be attributed to the 2*p*_3/2_ and 2*p*_1/2_ of Co, respectively, and the broad peaks located at 785.5 and 801.8 eV should be the typical satellite peaks of Co 2*p* [37,38,39]. In Figure 3f, the two peaks of the Cu 2*p* spectrum with binding energies of 932.3 and 952.1 eV could be respectively assigned to Cu 2*p*_3/2_ and Cu 2*p*_1/2_. Furthermore, binding energies of 943.2 and 962.4 eV were also observed relating to the two satellite peaks [14]. The two satellite peaks of 932.3 eV (CuO 2*p*_3/2_) and 952.1 eV (CuO 2*p*_1/2_) also verified the existence of Cu^2+^, indicating that the copper ions are mainly in the form of Cu^2+^ ions. These ions were fixed in the octahedral positions, enabling a more stable crystal structure [39]. Thus, based on the above results, the results of EDX and XPS measurements favorably confirmed the XRD results showing the formation of CuCo_2_O_4_ decoration on the CNFs.

### 3.2. Electrochemical Characterization of PTBA/CuCo_2_O_4_–CNFs/GCE

**Figure 4:**
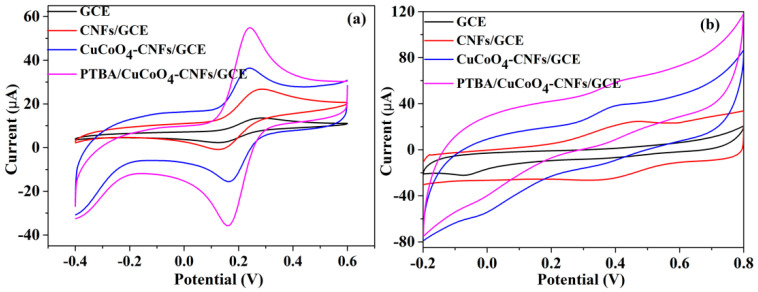
CV of bare GCE, CNFs/GCE, CuCo_2_O_4_–CNFs/GCE, and PTBA/CuCo_2_O_4_-CNFs/GCE in (**a**) 1.0 mM [Fe(CN)_6_]^3−/4−^ and (**b**) after accumulation of 2 mM glucose in 0.1 M NaOH at a scan rate of 50 mV/s.

As a valuable tool for the redox reaction of an electrochemical sensor, the electrochemical response of electroactive species [Fe(CN)6]^3−/4−^ was used to detect the dynamics of the interface barrier. Figure 4a gives the CV of [Fe(CN)6]^3−/4−^ at each step obtained on bare GCE, CNFs/GCE, CuCo_2_O_4_–CNFs/GCE, and PTBA/CuCo_2_O_4_–CNFs/GCE. For bare GCE, almost no significant anode and cathode peak currents were detected with a peak-to-peak distance (ΔEp) of 90 mV. After the electrode modification with CNFs, CNFs/GCE displayed two well-shaped redox peaks, accompanied with the enhancement of redox peak current, the decrease of peak separation value (85 mV), and the negative shift of peak potentials. The improvement of electrocatalytic response mainly contributed to the fast electron transfer derived from the three-dimensional network structure of CNF with good conductivity. After further decorating CNFs with CuCo_2_O_4_ nanoparticles, the CuCo_2_O_4_–CNFs/GCE electrode displayed significant electrocatalytic activity and excellent redox reversibility. These results can be explained as follows: when CuCo_2_O_4_ was incorporated with CNFs, the three-dimensional (3D) porous structure of CNFs with a large effective surface area provided high electrical conductivity, abundant nucleation sites, and dispersibility for CuCo_2_O_4_ decoration, while the mixed valence copper and cobalt in CuCo_2_O_4_ nanoparticles possessed high electrocatalytic activity as well. As for PTBA/CuCo_2_O_4_–CNFs/GCE, we used TBA for electrochemical polymerization and immobilization on the surface of CuCo_2_O_4_–CNFs/GCE with the further increased reversible redox peak current. It is well known that PTBA with excellent electrochemical performance has broad applications in the fields of electrocatalysis and electroanalysis [40]. The CV current response of [Fe(CN)_6_]^3−/4−^ on PTBA/CuCo_2_O_4_–CNFs/GCE was much higher than that of the CuCo_2_O_4_–CNFs/GCE and CNFs/GCE. This phenomenon could be attributed to the fact that the incorporation of PTBA onto the nanofiber substrate could improve the electrical conductivity of the composite and increase the reversibility of the [Fe(CN)_6_]^3−/4−^ redox reaction, thereby achieving a higher electrochemical response [41].

The electrocatalytic performance of the as-obtained electrodes toward glucose sensing in the alkaline medium was characterized by the CV technique. Figure 4b displays the CVs of different modified electrodes after accumulation of 2 mM glucose in 0.1 M NaOH. It can be seen that, in contrast to the bare GCE, the corresponding oxidation current of CNFs/GCE toward glucose oxidation increased significantly. This could be associated with the improved conductivity for CNFs along with the fast electron transport/collection. After CuCo_2_O_4_–CNF-modified electrode accumulation in 2 mM glucose, a significant anodic oxidation peak appeared around 0.46 V with the increased response current. As illustrated in Scheme 1b, the electrocatalytic process can be explained by the glucose being irreversibly oxidized to gluconic acid. As well known, nanostructure CuCo_2_O_4_, with the characteristics of large reachable surface area and short ion diffusion length, could effectively form Cu(III) and Co(III) strong oxidants in alkaline conditions, which participate in the oxidation of glucose to glucose lactone during the electron transfer reaction. Furthermore, the CNFs as matrices modified by CuCo_2_O_4_ NPs have the unique advantages of a one-dimensional nanostructure and effective nitrogen doping, which is beneficial to the diffusion and collection of conductive ions and electrons. Meanwhile, the rough surface structure of CNFs provides a sufficient interface contact between CuCo_2_O_4_ NPs and electrolytes. Moreover, a considerable improvement of sensitivity took place when TBA was electropolymerized onto CuCo_2_O_4_–CNFs/GCE, where the electrocatalytic response of PTBA/CuCo_2_O_4_–CNFs/GCE was obviously enhanced, showing sensitivity 1.5 times higher than that of CuCo_2_O_4_–CNFs/GCE. This demonstrated that the molecular reconstitution on the PTBA layer might provide an effective adsorption function for glucose immobilization, coupled with the rapid and sensitive electrode kinetic redox reaction. Thus, the PTBA and CuCo_2_O_4_–CNFs could be employed as outstanding catalysts for the electrocatalytic oxidation of glucose. Notably, the PTBA with borate recognition groups acts critically in the selective binding of carbohydrates via the formation of *cis*-diol bonding. Reversible complexes can be formed on the surface of modified electrodes by reversible covalent or electrostatic interactions under mild experiment conditions [42]. Similar phenomena were also reported in previous literature, where applying boronic acid group-bearing host compounds participated in a boronic acid–sugar specific bonding interaction. After a combination with metallic particles and various electrodes, the modified electrodes could be developed for selective capture and separation of *cis*-diol-containing biomolecules, such as epinephrine [40], cholesterol [41], uric acid [43], etc. This boronic acid-based immobilization modification approach offers a feasible selective environment for the target molecule, helping to further research and fabricate high-performance sensors. In this work, the molecular recognition property of the PTBA layer not only acts as the specific binding component for glucose sensing, but also benefits the enhancement of electrochemical properties because of its high conductibility. The results demonstrated the remarkable electro-catalysis performance of the hybrid sensor, mainly due to the synergistic effect of CNFs, CuCo_2_O_4_, and PTBA.

**Figure 5:**
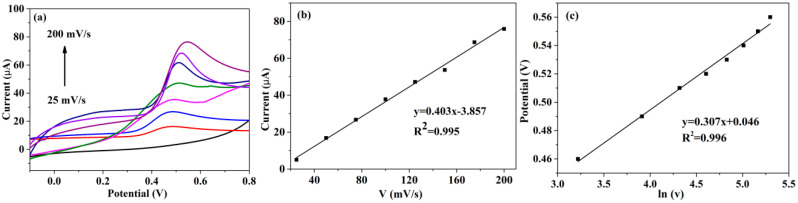
(**a**) CVs of PTBA/CuCo_2_O_4_–CNFs/GCE in 0.1 M NaOH with 1 mM glucose at scan rates ranging from 25 to 200 mV·s^−1^; (**b**) the linear regression of scan rate versus current signal; (**c**) the plots of peak potential with respect to ln ν.

The reaction kinetics of PTBA/CuCo_2_O_4_–CNFs/GCE was investigated as a function of scan rates after accumulation in 1 mM glucose. In Figure 5, the current response of glucose was enhanced with increasing scan rates from 25 to 200 mV·s^−1^. Moreover, the response current increased linearly with the increase in scan rate, with the linear relation expressed as Ipa (μA) = 0.403v − 3.857 (*R*^2^ = 0.995), implying that an adsorption-controlled electrochemical reaction process occurred on the modified electrodes. Moreover, the anodic peaks positions (Ep) were shifted to positive values with the increment of scan rate. The regression equation toward the dependence of Ep with ln v could be expressed as Ep (V) = 0.307 ln v + 0.046 (*R*^2^ = 0.996). The transferred electrons during the electrochemical catalytic oxidation process are based on the following equation [44]:

Ip = nFAv/4RT,(1)

where A is the peak area (known quantity) of a certain scan rate, R is the gas constant (8.314 J·mol^−1^·K^−1^), T is the temperature (300 K), and F is the Faraday constant (96,485 C·mol^−1^); v and Ip are the certain scanning rate and the corresponding peak current, respectively. Therefore, n was calculated to be 2.1; thus, the whole electrocatalytic process of glucose involved two electrons participating in the procedure. The detailed reaction mechanism of the detection is illustrated in Scheme 1b. In order to further determine the reaction mechanism of the electrocatalytic process, CV curves of the modified electrodes with and without glucose were investigated. As shown in Figure S1 (Supplementary Materials), CV curves of PTBA/CuCo_2_O_4_–CNFs/GCE in glucose concentrations of 0 mM and 3 mM were examined. The observed wide peaks of current relating to the anodic/cathodic peaks of Cu(III)/Cu(II) redox couples potential were obtained in the CV curve when no glucose was added [45]. Meanwhile, the redox peaks located at 0.38/0.65 V (III/IV) were mainly attributed to the effective participation of Co_3_O_4_ and CoOOH with reversible transition [46]. The involved electrochemical reactions toward Cu and Co elements in an alkaline environment are given in Equations (2) and (3) [47]. After the addition of glucose, the corresponding response current significantly increased. This was mainly due to the electrocatalytic action of the modified electrode with the oxidation of glucose to gluconolactone, while CoO_2_ was converted to CoOOH, and MOOH was converted to M(OH)_2_ (M = Cu, Co). The involved oxidation reaction mechanism took place, and this can be described by Equations (4) and (5) [46]. In addition, as the glucose concentration increased, the potential of the oxidation current shifted to a positive position, implying that the glucose oxidation intermediate product and glucose were effectively adsorbed at the active site of the modified electrode [48].

CuCo_2_O_4_ + OH^−^ + H_2_O ↔ CuOOH + 2CoOOH + e^−^. (2)

CoOOH + OH^−^ ↔ CoO_2_ + H_2_O + e^−^. (3)

2CoO_2_ +glucose → 2CoOOH + gluconolactone. (4)

2MOOH + glucose → 2M(OH)_2_ + gluconolactone. (5)

### 3.3. DPV Responses of PTBA/CuCo_2_O_4_–CNFs/GCE toward Glucose

**Figure 6:**
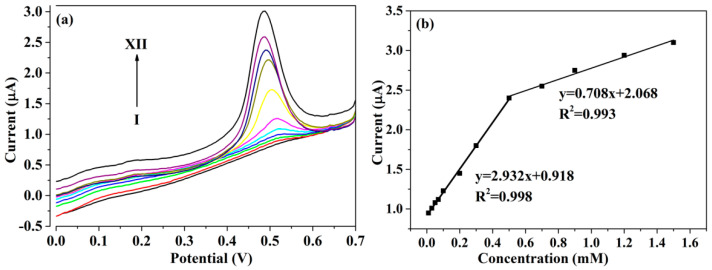
(**a**) Differential pulse voltammetry (DPV) measurements of PTBA/CuCo_2_O_4_–CNFs/GCE in 0.1 M NaOH after accumulation in different concentrations of glucose in the range of 0.01–1.5 mM; (**b**) the linear plots of current versus glucose concentration.

To enhance the sensitivity of the fabricated sensor, the differential pulse voltammetry technique (DPV) with excellent sensitivity was used to detect different concentrations of glucose. In Figure 6, the corresponding current responses of as-prepared PTBA/CuCo_2_O_4_–CNFs/GCE are recorded in an alkaline medium with the potential range of 0–0.7 V under the optimization conditions (Figure 6a). As shown in Figure 6b, the typical peak current–concentration response curve shows a dual linear region ranging from 0.01–0.5 mM with a linear regression of Ip (μA) = 2.932c + 0.918 (*R*^2^ = 0.998) and 0.5–1.5 mM with a linear regression of Ip (μA) = 0.708c + 2.068 (*R*^2^ = 0.993). The detection limit was estimated to be 0.15 μM (S/N = 3). The above results demonstrate that the as-fabricated PTBA/CuCo_2_O_4_–CNFs/GCE with favorable electrocatalytic properties could be used for the determination of glucose. Moreover, the PTBA/CuCo_2_O_4_–CNFs/GCE revealed superior analytical performance toward enzyme-free glucose detection compared with the previous literature. As shown in Table 1, our results are comparable to or stronger than those obtained other glucose sensors. In particular, the improved electrode has a lower detection limit than other sensors. This is mainly due to the low background current of the DPV detection method; thus, the DPV method has higher detection sensitivity and a lower detection limit in comparison with the widely used application method of amperometry. This demonstrates that the capability of the fabricated sensor for glucose detection was significantly improved.

### 3.4. Interference Studies

Anti-interference is a significant indicator for evaluating the practicability of the proposed non-enzymatic electrochemical sensors, because it is necessary for non-enzymatic electrochemical glucose sensors to be equipped with the ability to distinguish target analytes from interfering substances, as they commonly have a similar physiological environment. Some common interfering substances, such as AA, UA, DA, l-lysine, and NaCl, always coexist in real human blood serum, and they were employed for researching the interference effect. Table 2 reveals the results of interference experiments of those coexisting compounds on the response of PTBA/CuCo_2_O_4_–CNFs/GCE toward 2 mM glucose. It is evident that the interference response of PTBA/CuCo_2_O_4_–CNFs/GCE containing AA, UA, l-lysine, and NaCl can be almost neglected. Notably, the obtained current response toward DA was inconspicuous, which would not result in a noticeable effect on glucose detection. Therefore, the constructed sensing platform of PTBA/CuCo_2_O_4_–CNFs/GCE could successfully distinguished glucose from other coexisting interfering species with good accuracy in real sample applications.

The desirable selectivity of the as-prepared electrodes can be attributed to several aspects. Firstly, the nafion solution as an electrode stabilizer dropped on the surface was beneficial to the selectivity enhancement of the sensor, because the anionic polymer nafion could prevent some interfering substances from entering the electrode surface via electrostatic repulsion, such as interferants AA, UA, DA, etc., which are usually dissociated and negatively charged in NaOH solution [16]. In addition, CuCo_2_O_4_–CNFs possessed a three-dimensional porous nanostructure together with the outstanding electrocatalytic capacity of CuCo_2_O_4_, which is in favor of the fast adsorption of glucose and subsequent efficient diffusion to catalytically active sites. Therefore, in comparison with diffusion-controlled processes, adsorption-related reactions show minimal diffusion resistance; thus, they are helpful for the electrocatalytic detection of glucose [54]. Finally, PTBA films as a boronic acid-based host polymer compound were employed as the recognition unit because of their intense interactions with diol-containing compounds (i.e., glucose), which also contributed to the highly sensitive capability of the fabricated sensor.

### 3.5. Reproducibility, Stability, and Application for Real Samples

**Figure 7:**
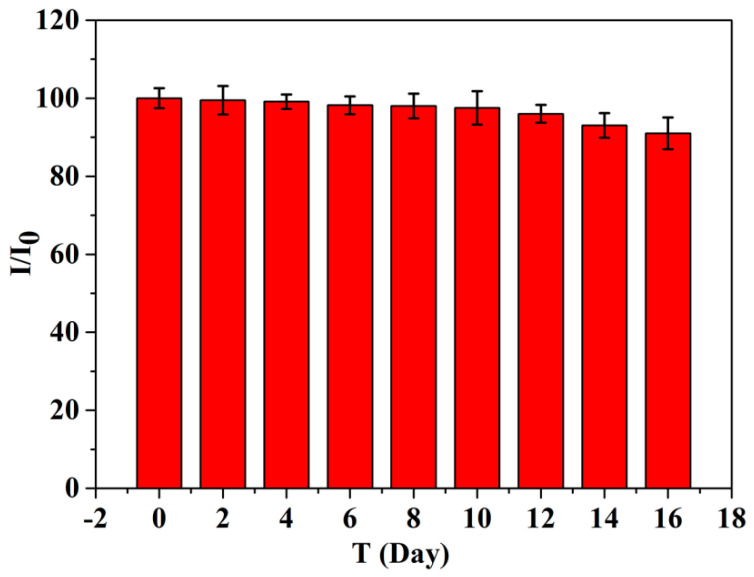
The stability of PTBA/CuCo_2_O_4_–CNFs/GCE sensor in the presence of 1 mM glucose after storage for more than two weeks.

To investigate the reproducibility of the fabricated electrode, six different PTBA/CuCo_2_O_4_–CNF-modified electrodes with the same preparation process were fabricated to perform an assessment in the presence of 2.0 mM glucose. All electrode response current values varied less with an RSD of 3.58%. Additionally, a RSD of 3.12% was obtained for five successive detections of 1.0 mM glucose with PTBA/CuCo_2_O_4_–CNF-modified electrodes, specifying the good stability of the fabricated sensor. The modified electrode was placed in the refrigerator to detect the long-term stability with the addition of 2 mM glucose at intervals of every two days. As shown in Figure 7, the electrochemical response of the obtained sensor did not change significantly, and 91% of the initial current response was maintained even after two weeks, mainly due to the structural and intrinsic stability of the CuCo_2_O_4_–CNFs and PTBA. Thus, the sensor could be applied in long-term practical applications.

Close monitoring of glucose in serum is an important means of controlling diabetes-related diseases. To certify the applicability of the sensor in real samples, the constructed PTBA/CuCo_2_O_4_–CNFs/GCE was applied to determine glucose in human blood samples. Before testing, the tested samples were diluted to various concentrations with 0.1 M PBS (phosphate buffer solution) solution. The corresponding DPV current responses were determined under optimal conditions in the potential range of 0–0.7 V. Certain concentrations of glucose were sequentially added to the serum, and then the corresponding response currents were recorded. The recoveries for standard added glucose were in the range of 98.5–105% with an RSD of 2.25–3.49% (Table 3), illustrating the feasibility of the proposed sensor system in real samples.

## 4. Conclusions

In summary, a three-dimensional network of CuCo_2_O_4_–CNFs was fabricated successfully via an in situ electrospinning technique and a subsequent carbonizing process. A large number of CuCo_2_O_4_ nanoparticles uniformly anchored onto the surface of carbon nanofibers with the characteristics of high specific surface area, 3D heterogeneous network structure, and excellent electrical conductivity, which contributed to the plentiful active sites and high electrocatalytic activity. The specific interactions between the glucose and electrode materials were achieved by electrochemical polymerization of thiophene-3-boronic acid (TBA). Benefiting from the selective recognition pattern of boronic acid groups, glucose molecules could be pre-deposited onto the surface of the hybrid electrode interface via reversible covalent and electrostatic interactions. The obtained PTBA/CuCo_2_O_4_–CNF composites exhibited outstanding electrocatalytic performance toward glucose and demonstrated excellent sensitivity, with a wide detection range and a low detection limit. Therefore, the facile synthesis of hybrid nanofiber materials is promising for constructing a novel non-enzymatic glucose sensor for determining real serum samples in the future.

## Figures and Tables

**Table 1:** Comparison of the performance of PTBA/CuCo_2_O_4_–CNFs/GCE sensor with other non-enzymatic glucose sensors.

Materials	Method	Sensitivity (µA·cm^−2^·mM^−1^)	Linear Range (mM)	Detection Limit (µM)	Reference
CuO-PANI	Amperometry	28001359	0.00025–0.28 0.28–4.6	0.24	[49]
NiCo_2_O_4_/ECF	Amperometry	1947.2	0.005–19.175	1.5	[50]
CuCo_2_O_4_/PrGO	Amperometry	2426	0.0005–3.354	0.15	[51]
CuCo_2_O_4_ nanosheets	Amperometry	3.625	Up to 0.32	5	[36]
NiONFs–rGO	Amperometry	1100	0.002–0.6	0.77	[52]
Co_3_O_4_@PANINFs	Amperometry	14.25	0.1–8	60	[53]
PTBA/CuCo_2_O_4_–CNFs	DPV	2932708	0.01–0.50.5–1.5	0.15	This work

**Table 2:** Results of interference experiments on the current intensity of 2 mM glucose on PTBA/CuCo_2_O_4_–CNFs/GCE.

Interference	Concentration (mM)	Current Ratio	RSD (%)
Ascorbic acid (AA)	2	0.99	2.35
Uric acid (UA)	2	0.98	1.59
Dopamine (DA)	1	0.93	3.56
Sodium chloride	10	0.99	1.28
l-Lysine	2	0.98	2.69

**Table 3:** Analysis of glucose in human serum samples using PTBA/CuCo_2_O_4_–CNFs/GCE.

No.	Added (mM)	Founded (mM)	Recovery (%)	RSD (%)
1	0.3	0.31	103	2.25
2	0.7	0.69	98.5	3.49
3	1.2	1.26	105	2.72

## Supplementary Materials

The following are available online at https://www.mdpi.com/1996-1944/13/12/2874/s1: Figure S1. CVs of in PTBA/CuCo2O4-CNFs/GCE in 0.1 M NaOH solution in the absence (black) and presence (red) of 2 mM glucose with the scan rate of 20 mV·s^−1^.
