# Supplementary material for: A Nonenzymatic Glucose Sensor Platform Based on Specific Recognition and Conductive Polymer-Decorated CuCo2O4 Carbon Nanofibers
Source: Materials (Basel). 2020 Jun 26;13(12):2874. doi: 10.3390/ma13122874 (PMC7345228; doi:10.3390/ma13122874)
Supplement: Supplementary file 1 [file materials-13-02874-s001.pdf]

A Nonenzymatic Glucose Sensor Platform Based on Specific Recognition and Conductive Polymer-Decorated CuCo2O4 Carbon Nanofibers

Yongling Ding[1,2,3,4,*], Huadong Sun[1,*], Chunrong Ren[1], Mingchen Zhang[3] and Kangning Sun [4,*]

[1]School of Transportation Civil Engineering, Shandong Jiaotong University, Jinan 250357, China; 204130@sdjtu.edu.cn (Y.D.); 204113@sdjtu.edu.cn (H.S.); 214052@sdjtu.edu.cn

[2]School of Control Science and Engineering, Shandong University, Jinan 250002, China

[3]Postdoctoral Technology Research Center, Shandong Anran Group, Weihai 264205, China; zhangmingchen@126.com

[4]School of Materials Science and Engineering, Shandong University, Jinan 250002, China

[*]Correspondence: 204130@sdjtu.edu.cn (Y.D.); 204113@sdjtu.edu.cn (H.S.); sunkangning@sdu.edu.cn (K.S.)

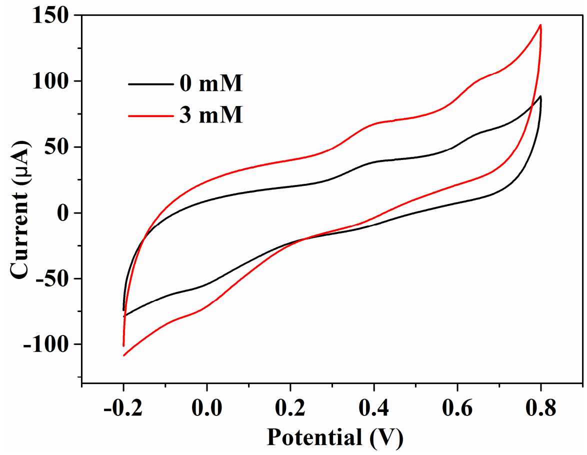

Figure S1 CVs of in PTBA/$CuCo_2O_4$-CNFs/GCE in 0.1 M NaOH solution in the absence (black) and presence (red) of 2 mM glucose with the scan rate of 20 mV·s^{-1}.
